# Editorial: Advances in combating antimicrobial resistance: focus on diagnosis, therapy, and prevention

**DOI:** 10.3389/fcimb.2026.1889025

**Published:** 2026-06-18

**Authors:** Yuetian Yu, Jiabao Xu, Bin Lin, Tatiana V. Priputnevich, Muhammad Usman Munir, Sue C. Nang, Zhongheng Zhang

**Affiliations:** 1 Department of Critical Care Medicine, Renji Hospital, School of Medicine, Shanghai Jiao Tong University, Shanghai, China; 2 Key Laboratory of Intelligent Pharmacy and Individualized Therapy of Huzhou, Huzhou, China; 3 James Watt School of Engineering, University of Glasgow, Glasgow, United Kingdom; 4 Department of Pharmacy, Changxing People’s Hospital, Second Affiliated Hospital of Zhejiang University School of Medicine, Huzhou, China; 5 Institute of Microbiology, Antimicrobial Therapy and Epidemiology, Federal State Budgetary Institution “National Medical Research Center for Obstetrics, Gynecology and Perinatology named after V.I. Kulakov” of the Ministry of Health of the Russian Federation, Moscow, Russia; 6 Department of Medical Microbiology, Federal State Budgetary Educational Institution of Further Professional Education «Russian Medical Academy of Continuous Professional Education» of the Ministry of Health of the Russian Federation, Moscow, Russia; 7 Australian Institute for Bioengineering and Nanotechnology, The University of Queensland, Brisbane, QLD, Australia; 8 Department of Microbiology, Biomedicine Discovery Institute, Monash University, Clayton, VIC, Australia; 9 Department of Emergency Medicine, Sir Run-Run Shaw Hospital, Zhejiang University School of Medicine, Hangzhou, China; 10 Zhejiang Key Laboratory of Precise Diagnosis and Treatment of Abdominal Infection, Sir Run Run Shaw Hospital, Zhejiang University School of Medicine, Hangzhou, China; 11 School of Medicine, Shaoxing University, Shaoxing, China; 12 Key Laboratory of Emergency and Trauma of Ministry of Education, Engineering Research Center for Hainan Biological Sample Resources of Major Diseases, The First Affiliated Hospital, Hainan Medical University, Haikou, China

**Keywords:** antimicrobial resistance, antimicrobial stewardship, multidrug-resistant bacteria, novel therapeutics, One Health, rapid diagnostics

## The escalating threat of antimicrobial resistance: global and ICU epidemiology

Antimicrobial resistance (AMR) has emerged as one of the most pressing public health threats of the 21^st^ century. The World Health Organization (WHO) has projected that without effective interventions, AMR could claim 10 million lives annually by 2050, surpassing cancer as a leading cause of death and imposing a cumulative economic burden of $100 trillion globally (GBD 2021 Antimicrobial Resistance Collaborators, 2024). The clinical landscape of AMR is particularly dire in intensive care units (ICUs), where the convergence of critically ill patients, extensive use of invasive devices, and high selective pressure from broad-spectrum antibiotics creates an ideal environment for the emergence and dissemination of multidrug-resistant organisms (MDROs) and carbapenem-resistant organisms (CROs) (Ture et al., 2022; Zhu et al., 2025).

Infections caused by carbapenem-resistant *Enterobacteriaceae* (CRE), carbapenem-resistant *Acinetobacter bauman*nii (CRAB), carbapenem-resistant *Pseudomonas aeruginosa* (CRPA), and vancomycin-resistant *Enterococci* (VRE) are associated with devastating clinical outcomes (Zhong et al., 2021; Jin et al., 2025). A systematic analysis from the GBD 2021 Antimicrobial Resistance Collaborators revealed that bacterial AMR directly caused 1.14 million deaths in 2021 and was associated with 4.71 million deaths globally (GBD 2021 Antimicrobial Resistance Collaborators, 2024). In ICU settings, patients with CRO infections experience prolonged mechanical ventilation, extended ICU and hospital stay (often exceeding 20–30 days), exorbitant healthcare costs (2–3 times higher than susceptible infections), and mortality rates ranging from 40% to over 60% for CRE and CRAB infections (Li et al., 2019; Zhen et al., 2020; Zhang et al., 2026).

The health economic burden is also staggering. A recent multicenter study across 10 countries estimated that the excess cost per CRO infection ranges from $20,000 to $120,000, driven by the need for combined use of broad-spectrum antibiotics, prolonged use of extracorporeal life support devices, enhanced infection control measures, and prolonged rehabilitation (Poudel et al., 2023). Moreover, the erosion of last-line antibiotics has forced clinicians to rely on older, more toxic agents such as colistin and tigecycline, further compromising patient outcomes.

This Research Topic, *Advances in combating antimicrobial resistance: focus on diagnosis, therapy, and prevention*, brings together 19 articles from dozens of submissions that address this multifaceted crisis across three interdependent pillars. The contributions herein collectively illuminate a transition from reactive, empiric management to proactive, precision-based countermeasures.

## Diagnostic advances: rapid pathogen identification and resistance detection

A fundamental obstacle in AMR management has been the reliance on traditional culture-based methods, which require 48–72 hours for pathogen identification and an additional 24–48 hours for antimicrobial susceptibility testing (AST). This delay forces clinicians to initiate empiric broad-spectrum therapy, inadvertently driving further resistance. In response, rapid diagnostic tests (RDTs) have revolutionized the field, enabling same-day pathogen identification and resistance gene detection directly from clinical specimens. Meanwhile, the process of blood culture is constantly being improved to shorten turn around time (TAT) (Qin et al., 2025; Wang et al., 2025).

Several articles in this Research Topic showcase diagnostic innovations. Yu et al. developed a deep learning algorithm based on endoscopic features for rapid *Helicobacter pylori* infection status assessment, reducing reliance on multiple biopsies and culture confirmation. Zhang et al. employed machine learning to investigate factors influencing nirmatrelvir/ritonavir exposure, demonstrating how computational models can predict drug concentration variability-a principle translatable to resistance prediction. Zhang et al. characterized the molecular epidemiology of CRE in neonates from two large children’s hospitals in Southwestern China, identifying dominant resistance gene reservoirs that can guide targeted empiric therapy. Wu et al. established tentative epidemiological cutoff values for faropenem against key bacterial pathogens, offering a benchmark for detecting non-wild-type populations.

Beyond molecular panels, metagenomic next-generation sequencing (mNGS) and CRISPR-based assays represent the next frontier. mNGS enables unbiased detection of virtually almost all pathogens in a single test, proving invaluable for culture-negative infections, unexpected or fastidious pathogens, and polymicrobial communities (Wang et al., 2021; Wang et al., 2024). CRISPR-based diagnostics offer rapid, low-cost, point-of-care detection with high specificity. The application of these advanced diagnostics is moving us from educated guesswork to precise pathogen identification, forming the bedrock of modern antimicrobial stewardship. After nearly a decade of clinical application, mNGS has demonstrated its advantages over traditional detection methods in the rapid and accurate identification of pathogens. In the future, it will be further localized and efforts will be made to build multi-disciplinary teams (MDT) for the treatment of infections. Together, they will work to accurately interpret relevant reports, contributing to improved patient outcomes.

Parallel to rapid phenotypic profiling, understanding the spatial architecture of polymicrobial infections is critical, as standard diagnostic cultures often disrupt complex microbial communities. In this context, ribosomal fluorescence *in situ* hybridization (FISH) serves as an indispensable tool for the direct, culture-independent visualization of bacterial biofilms within host tissues. As pioneered by Swidsinski and colleagues, the deployment of sequence-specific fluorescent oligonucleotide probes enables the identification of structured, pathogen-specific consortia that otherwise evade conventional mono-infectious diagnostic paradigms (Swidsinski et al., 2024). By combining strict taxonomic identification with exact morphological localization, FISH-based diagnostic frameworks bypass the limitations of routine microbiology, offering crucial insights into biofilm-mediated resistance and guiding targeted, localized therapeutic interventions.

## Therapeutic advances: novel agents, optimized dosing and alternative strategies

Confronted with pan-resistant bacteria, replenishing the therapeutic pipeline is an urgent clinical necessity. This Research Topic presents real-world evidence on both established and emerging agents, as well as non-antibiotic strategies.

### Novel antimicrobial agents

The past decade has witnessed the introduction of several new β-lactam/β-lactamase inhibitor (BLBLI) combinations, including ceftazidime-avibactam, ceftolozane-tazobactam, meropenem-vaborbactam, imipenem-relebactam and sulbactam-durlobactam. These agents have demonstrated efficacy against CROs (MaChado et al., 2016). However, resistance to even these new agents has already emerged, underscoring the relentless adaptability of microbes (Prindle et al., 2015). Fang et al. provide a granular view of in-host co-colonization and bloodstream infection by distinct classical and hypervirulent carbapenem-resistant *Klebsiella Pneumonia*e (CRKP) clones harboring a homologous *blaKPC-2* plasmid, highlighting horizontal resistance transfer and the potential need for combination or sequential therapies.

### Non-antibiotic therapeutic strategies

Beyond traditional antibiotics, innovative approaches are gaining traction. Kart et al., in their mini-review, explore encapsulated probiotics as antimicrobial agents, detailing delivery mechanisms against MDR pathogens. This approach leverages microbiome modulation to combat infection while preserving beneficial commensals. Bacteriophage therapy, though not featured directly in this Research Topic, represents a long-considered option for untreatable infections. Phages are viruses that specifically lyse bacteria, offering narrow-spectrum activity that spares the microbiota. Similarly, monoclonal antibodies targeting bacterial toxins or surface antigens and immunomodulatory agents are promising adjunctive strategies that move therapy beyond direct microbicidal action to holistic host support (Millar et al., 2026). Understanding resistance-virulence co-evolution is critical for therapeutic development. Lin et al., in a comprehensive review, dissect the co-evolution of resistance and virulence in *K. pneumoniae* liver abscess, focusing on hypervirulent variants and the therapeutic dilemmas posed by concurrent carbapenem resistance and high virulence. These strains, often associated with the *rmpA* and *rmpA2* genes, cause invasive infections in healthy individuals and are increasingly reported worldwide.

To outpace the rapid evolution of MDR pathogens, the therapeutic pipeline must expand beyond classical bactericidal or bacteriostatic scaffolds toward targets less prone to conventional resistance mechanisms. Among these alternative strategies, disrupting prokaryotic ion channels represents a highly promising, yet relatively underexplored, paradigm. Bacterial mechanosensitive (MS) channels (such as *MscL* and *MscS*) and voltage-gated potassium/calcium transport systems are crucial for maintaining cellular turgor, pH homeostasis, and membrane potential. Pharmacological manipulation of these channels—either via the design of channel blockers that trigger osmotic or metabolic collapse, or through MscL agonists that induce uncontrolled leakage of essential cytoplasmic osmolytes—effectively cripples bacterial viability. Crucially, because these ion systems also regulate the energetic requirements of transmembrane efflux pumps, the application of ion channel blockers has been shown to synergistically reverse drug resistance by preventing the extrusion of co-administered conventional antibiotics and enhancing macrophage-mediated killing of intracellular pathogens (Tamma et al., 2024).

Beyond individual cellular homeostasis, targeting ion channel dynamics offers a powerful approach to dismantling complex bacterial biofilm architectures. It has been established that bacteria within biofilms utilize long-range electrical signaling, mediated by coordinated waves of potassium ion efflux, to communicate metabolic stress and synchronize defense behaviors between the interior and periphery of the community. By integrating ion channel modulators into anti-biofilm regimens, this electrochemical communication network can be effectively short-circuited, leaving the biofilm vulnerable to host immune clearance and standard antimicrobials (Garlasco et al., 2026). Complementing this biophysical approach, other non-antibiotic strategies featured in this Research Topic include the deployment of predatory bacteria, such as *Bdellovibrio bacteriovorus*, which act as “living antibiotics” to physically consume Gram-negative pathogens without triggering receptor-mediated resistance. Additionally, the use of engineered antimicrobial peptides (AMPs) combined with membrane-disrupting agents bypasses traditional metabolic resistance pathways altogether, highlighting a multifaceted future for non-traditional anti-infective discovery (Giamarellos-Bourboulis et al., 2024).

### Optimizing existing agents: PK/PD and TDM

Simply prescribing the right antibiotic is insufficient, achieving adequate exposure at the infection site is paramount. The pathophysiological alterations in sepsis augmented renal clearance (ARC), capillary leak, organ dysfunction, and extracorporeal circuits lead to highly variable antibiotic concentrations (Roberts et al., 2014). Therapeutic drug monitoring (TDM) of antibiotics, particularly for vancomycin and aminoglycosides, has been standard practice. This Research Topic expands this concept to β-lactams and other time-dependent antibiotics, demonstrating that a substantial proportion of critically ill patients fail to achieve target pharmacokinetic/pharmacodynamics (PK/PD) indices with standard dosing, and that TDM-guided adjustment improves clinical outcomes. Model-informed precision dosing (MIPD) using Bayesian algorithms represents the next leap forward, enabling individualized dosing from the outset rather than reactive adjustments.

## Preventive advances: stewardship, infection control and One Health

Prevention remains the most sustainable and cost-effective strategy to combat AMR. This Research Topic emphasizes multi-level prevention, from individual prescribers to global policy.

### Antimicrobial stewardship

AMS programs optimize antibiotic use to maximize clinical benefit while minimizing unintended consequences. Alsalhani et al. present a critical integration of One Health perspectives into dental antibiotic stewardship in Saudi Arabia, revealing that inappropriate prescribing in dentistry significantly contributes to community resistance. Their survey of 457 dentists found that 68% prescribed antibiotics for conditions not indicated by guidelines, highlighting an urgent target for stewardship expansion.

### Infection prevention and control

A systematic review and meta-analysis by Huang et al. on risk factors for CRKP infection synthesizes data from 28 studies (*n* = 12,847 patients). Key modifiable risk factors included prior antibiotic exposure (OR 3.42, 95% CI 2.18-5.36), ICU admission (OR 2.89, 95% CI 1.92-4.35), mechanical ventilation (OR 3.12, 95% CI 2.01-4.84), and central venous catheterization (OR 2.54, 95% CI 1.67-3.86). These findings enable targeted IPC measures, including admission screening for high-risk patients, enhanced environmental disinfection, and contact precautions. Zhao et al. highlight a specific high-risk population, demonstrating that multidrug-resistant *Clostridioides difficile* isolate ST81 is prominent in hematological patients (41.7% of isolates from this population). This finding advocates for routine screening and enhanced barrier precautions in immunocompromised hosts, where standard infection control may be insufficient.

### One Health and global policy

On a macro level, Mba and Martins provide a strategic roadmap for combating AMR in Africa, addressing gaps in surveillance, stewardship and research. Their blueprint emphasizes: (1) establishing national AMR surveillance networks aligned with WHO Global Antimicrobial Resistance Surveillance System (GLASS); (2) strengthening laboratory capacity for culture and AST; (3) implementing national action plans with dedicated funding; and (4) engaging community stakeholders to promote responsible antibiotic use in humans, animals and agriculture. The One Health approach recognizes that AMR circulates across human, animal and environmental reservoirs. Livestock antibiotic use, agricultural runoff and wildlife contamination all contribute to the resistance gene pool. Effective prevention requires integrated surveillance, antibiotic use regulation in food animals and public education campaigns.

### New preventive tools

Beyond traditional IPC, novel technologies are emerging. Air filtration systems with HEPA and UV-C light, copper-impregnated surfaces, and automated UV-C disinfection robots reduce environmental bioburden. Probiotic-based competitive exclusion strategies are being explored to prevent colonization with MDR pathogens in high-risk patients. Additionally, rapid molecular screening for asymptomatic carriage of CRE or methicillin-resistant *Staphylococcus aureus* (MRSA) on admission enables pre-emptive isolation and decolonization.

## Conclusion and future directions: toward multi-disciplinary, lifecycle AMR management

The 19 articles in this Research Topic collectively chart a future where AMR is met with a coordinated, technologically sophisticated counteroffensive across diagnosis, therapy and prevention. The traditional siloed approach, where diagnostics, therapeutics and prevention operate independently, is giving way to an integrated, multi-disciplinary, lifecycle management model. Key future directions include five aspects: (1) Integrated diagnostic-therapeutic algorithms - Real-time integration of RDTs results with electronic health records and clinical decision support systems to guide targeted therapy within hours. (2) Accelerated therapeutic development - Prioritization of antibiotics targeting WHO priority pathogens, leveraging artificial intelligence for drug discovery, and streamlining regulatory pathways for non-traditional agents. (3) Personalized prevention - Risk stratification using clinical prediction models and rapid admission screening to target IPC measures to high-risk patients, maximizing efficiency and minimizing isolation burden. (4) Global AMR surveillance - Expansion of GLASS and regional networks to provide real-time resistance data, enabling early detection of emerging resistance and guiding empiric therapy recommendations. (5) One Health implementation - Operationalizing the One Health framework through cross-sectoral governance, shared antimicrobial use targets, and integrated surveillance. Additionally, education campaigns are needed to reduce inappropriate antibiotic demand, improve adherence, and promote prevention.

The journey ahead requires collaboration among intensivists, infectious disease physicians, microbiologists, clinical pharmacists, data scientists, infection control practitioners, and policymakers. The work presented in this Research Topic is a testament to the vibrant and innovative spirit of this community. By embracing and integrating these advancements, we can transform the fight against AMR from a reactive struggle into a proactive, precise and sustainable endeavor, ensuring that effective antibiotics remain available for current and future generations.

## References

[B1] Garlasco J. Arieti F. Morra M. Tebon M. Ortiz D. Pezzani M. D. . (2026). The Emerging Resistance Index: tracking early resistance to new antibiotics. Lancet Infect. Dis. 26, e219–e227. doi: 10.1016/S1473-3099(25)00501-8 41067238

[B2] GBD 2021 Antimicrobial Resistance Collaborators (2024). Global burden of bacterial antimicrobial resistance 1990-2021: a systematic analysis with forecasts to 2050. Lancet 404, 1199–1226. doi: 10.1016/S0140-6736(24)01867-1 39299261 PMC11718157

[B3] Giamarellos-Bourboulis E. J. Aschenbrenner A. C. Bauer M. Bock C. Calandra T. Gat-Viks I. . (2024). The pathophysiology of sepsis and precision-medicine-based immunotherapy. Nat. Immunol. 25, 19–28. doi: 10.1038/s41590-023-01660-5 38168953

[B4] Jin S. S. Wang W. Q. Jiang Y. H. Yu Y. T. Wang R. L. (2025). A comprehensive overview of Klebsiella pneumoniae: resistance dynamics, clinical manifestations, and therapeutic options. Infect. Drug Resist. 18, 1611–1628. doi: 10.2147/IDR.S502175 40162036 PMC11954396

[B5] Li Y. Shen H. Zhu C. Yu Y. (2019). Carbapenem-resistant Klebsiella pneumoniae infections among ICU admission patients in Central China: prevalence and prediction model. BioMed. Res. Int. 2019, 9767313. doi: 10.1155/2019/9767313 31032370 PMC6457282

[B6] MaChado D. Pires D. Perdigão J. Couto I. Portugal I. Martins M. . (2016). Ion channel blockers as antimicrobial agents, efflux inhibitors, and enhancers of macrophage killing activity against drug resistant Mycobacterium tuberculosis. PloS One 11, e0149326. doi: 10.1371/journal.pone.0149326 26919135 PMC4769142

[B7] Millar B. C. Cates M. J. Torrisi M. S. Round A. J. Warde A. Lowery C. J. . (2026). Antimicrobial resistance: the answers. Br. J. Biomed. Sci. 83, 15559. doi: 10.3389/bjbs.2026.15559 41727556 PMC12922517

[B8] Poudel A. N. Zhu S. Cooper N. Little P. Tarrant C. Hickman M. . (2023). The economic burden of antibiotic resistance: a systematic review and meta-analysis. PloS One 18, e0285170. doi: 10.1371/journal.pone.0285170 37155660 PMC10166566

[B9] Prindle A. Liu J. Asally M. Ly S. Garcia-Ojalvo J. Süel G. M. (2015). Ion channels enable electrical communication in bacterial communities. Nature 527, 59–63. doi: 10.1038/nature15709 26503040 PMC4890463

[B10] Qin J. Zhang H. Zhang X. Wang L. Yu Y. Li M. . (2025). Optimizing blood culture diagnostics through laboratory automation: reducing turnaround time and improving clinical outcomes. Microbiol. Spectr 13, e0192725. doi: 10.1128/spectrum.01927-25 41217182 PMC12671119

[B11] Roberts J. A. Abdul-Aziz M. H. Lipman J. Mouton J. W. Vinks A. A. Felton T. W. . (2014). Individualised antibiotic dosing for patients who are critically ill: challenges and potential solutions. Lancet Infect. Dis. 14, 498–509. doi: 10.1016/S1473-3099(14)70036-2 24768475 PMC4181663

[B12] Swidsinski A. Amann R. Guschin A. Swidsinski S. Loening-Baucke V. Mendling W. . (2024). Polymicrobial consortia in the pathogenesis of biofilm vaginosis visualized by FISH. Historic review outlining the basic principles of the polymicrobial infection theory. Microbes Infect. 26, 105403. doi: 10.1016/j.micinf.2024.105403 39127090

[B13] Tamma P. D. Heil E. L. Justo J. A. Mathers A. J. Satlin M. J. Bonomo R. A. (2024). Infectious Diseases Society of America 2024 guidance on the treatment of antimicrobial-resistant Gram-negative infections. Clin. Infect. Dis., ciae403. doi: 10.1093/cid/ciae403 39108079

[B14] Ture Z. Güner R. Alp E. (2022). Antimicrobial stewardship in the intensive care unit. J. Intensive Med. 3, 244–253. doi: 10.1016/j.jointm.2022.10.001 37533805 PMC10391567

[B15] Wang L. Guo W. Shen H. Guo J. Wen D. Yu Y. . (2021). Plasma microbial cell-free DNA sequencing technology for the diagnosis of sepsis in the ICU. Front. Mol. Biosci. 8, 659390. doi: 10.3389/fmolb.2021.659390 34124149 PMC8194294

[B16] Wang L. Lin B. Gong X. Yu Y. (2025). Editorial: Progressing the understanding and management of bloodstream infections. Front. Med. Lausanne 12, 1562934. doi: 10.3389/fmed.2025.1562934 39967598 PMC11832539

[B17] Wang L. Tian W. Zhang W. Wen D. Yang S. Wang J. . (2024). A machine learning model for predicting sepsis based on an optimized assay for microbial cell-free DNA sequencing. Clin. Chim. Acta 559, 119716. doi: 10.1016/j.cca.2024.119716 38710402

[B18] Zhang J. Zhou R. Ren J. Wu Z. Shen J. Wang Y. . (2026). Economic burden of carbapenem-resistant Klebsiella pneumoniae infections in Chinese hospitals: a 2019 analysis. J. Glob. Antimicrob. Resist. 46, 63–70. doi: 10.1016/j.jgar.2025.11.006 41260519

[B19] Zhen X. Stålsby Lundborg C. Sun X. Gu S. Dong H. (2020). Clinical and economic burden of carbapenem-resistant infection or colonization caused by Klebsiella pneumoniae, Pseudomonas aeruginosa, Acinetobacter baumannii: a multicenter study in China. Antibiotics Bsl 9, 514. doi: 10.3390/antibiotics9080514 32823707 PMC7459498

[B20] Zhong H. Chen F. Li Y. J. Zhao X. Y. Zhang Z. L. Gu Z. C. . (2021). Global trends and hotspots in research of carbapenem-resistant Enterobacteriaceae (CRE): a bibliometric analysis from 2010 to 2020. Ann. Palliat Med. 10, 6079–6091. doi: 10.21037/apm-21-87 34237952

[B21] Zhu C. Lin B. Zhang Z. Yu Y. (2025). Editorial: Infections in the intensive care unit, volume III. Front. Med. Lausanne 12, 1719505. doi: 10.3389/fmed.2025.1719505 41244796 PMC12611745

